# Giant Facial Angiofibroma as an Unusual Manifestation of Tuberous Sclerosis Complex

**DOI:** 10.7759/cureus.85367

**Published:** 2025-06-04

**Authors:** Zonia Moore, Ilse Osorio-Aragón, Alberto Saba Mussali, Elisa Vega-Memije

**Affiliations:** 1 Department of Dermatology, Hospital General Dr. Manuel Gea González, Mexico City, MEX; 2 College of Medicine, University of Pennsylvania Perelman School of Medicine, Pennsylvania, USA; 3 Department of Dermatology and Oncology, Ladislao de la Pascua, Mexico City, MEX

**Keywords:** adenoma sebaceum, angiofibroma, gigantic angiofibroma, tuberous sclerosis, tuberous sclerosis complex

## Abstract

Tuberous sclerosis complex (TSC) is a genetic disease characterized by the growth of tuberous fibromas in various locations of the body, due to a mutation in the protein tuberin or hamartin. This mutation leads to significant neurological and functional impairment, as well as dysregulation in the mTOR pathway. We review the case of a 47-year-old Hispanic man born with TSC, who presented to our service with a large mandibular neoformation. He is non-verbal and presents with dental enamel pits, epilepsy, intellectual disability, and other manifestations of TSC. Resection of the neoformation was performed under localized anesthesia, and histopathology confirmed a diagnosis of angiofibroma, a gigantic one due to the overall size. This case is notable for the size of the neoformation, the location, and the rapid growth pattern presented. A foreign body reaction may compound the underlying mTORopathy to contribute to the pathogenesis of a giant angiofibroma. This case illustrates management principles for giant angiofibromas and elucidates a pathophysiologic mechanism for their development.

## Introduction

Tuberous sclerosis complex (TSC) is a neurocutaneous genetic disorder that affects multiple systems in the body. With an incidence of one per 6,000 to 10,000 live births, TSC is caused by a mutation in either the TSC1 gene, which encodes the protein hamartin, or the TSC2 gene, encoding tuberin [1,2]. Approximately 30% of TSC cases are inherited in an autosomal dominant manner, while 70% arise from spontaneous mutations. The most common dermatologic finding is “ash leaf spots” (hypomelanotic macules), present in 90% of people living with TSC. Some have a fibrous cephalic plaque typically located on the forehead or a shagreen patch on the lower back. Confetti skin lesions and ungual fibromas are also common. Up to 75% of people who live with TSC past the age of nine will develop an angiofibroma (previously known as an adenoma sebaceum). Angiofibromas are classically found on the nose and central face and grow slowly with time [2].

Previous research has shown that TSC1 and TSC2 mutations lead to dysregulation of the mTOR pathway. When the TSC1:TSC2 complex protein does not form, mTOR remains inactive and tissue overgrowth can occur. Dysregulation of this pathway has been pinned as the pathophysiology behind all tissue overgrowth in TSC, including angiofibroma formation [2]. We present the case of a rapidly enlarging giant angiofibroma located on the mandible instead of the central face. There is little research into the pathophysiology of gigantic angiofibroma occurring in uncommon locations with uncommon growth patterns. The case emphasizes the variability of angiofibroma growth patterns and raises awareness surrounding how to manage them, including prompt surgical management in adequate cases. Our aim is to provide insight into the pathophysiology and management challenges associated with such an atypical case of rapidly growing giant angiofibroma.

## Case presentation

Our patient is a 47-year-old Hispanic man who was diagnosed with TSC as an infant. During birth, he was subjected to a significant period of cerebral hypoxia. Consequently, he experiences near-intractable epileptic seizures and is treated with valproate, phenobarbital, phenytoin, and levetiracetam, resulting in a somnolent state.

**Figure 1:**
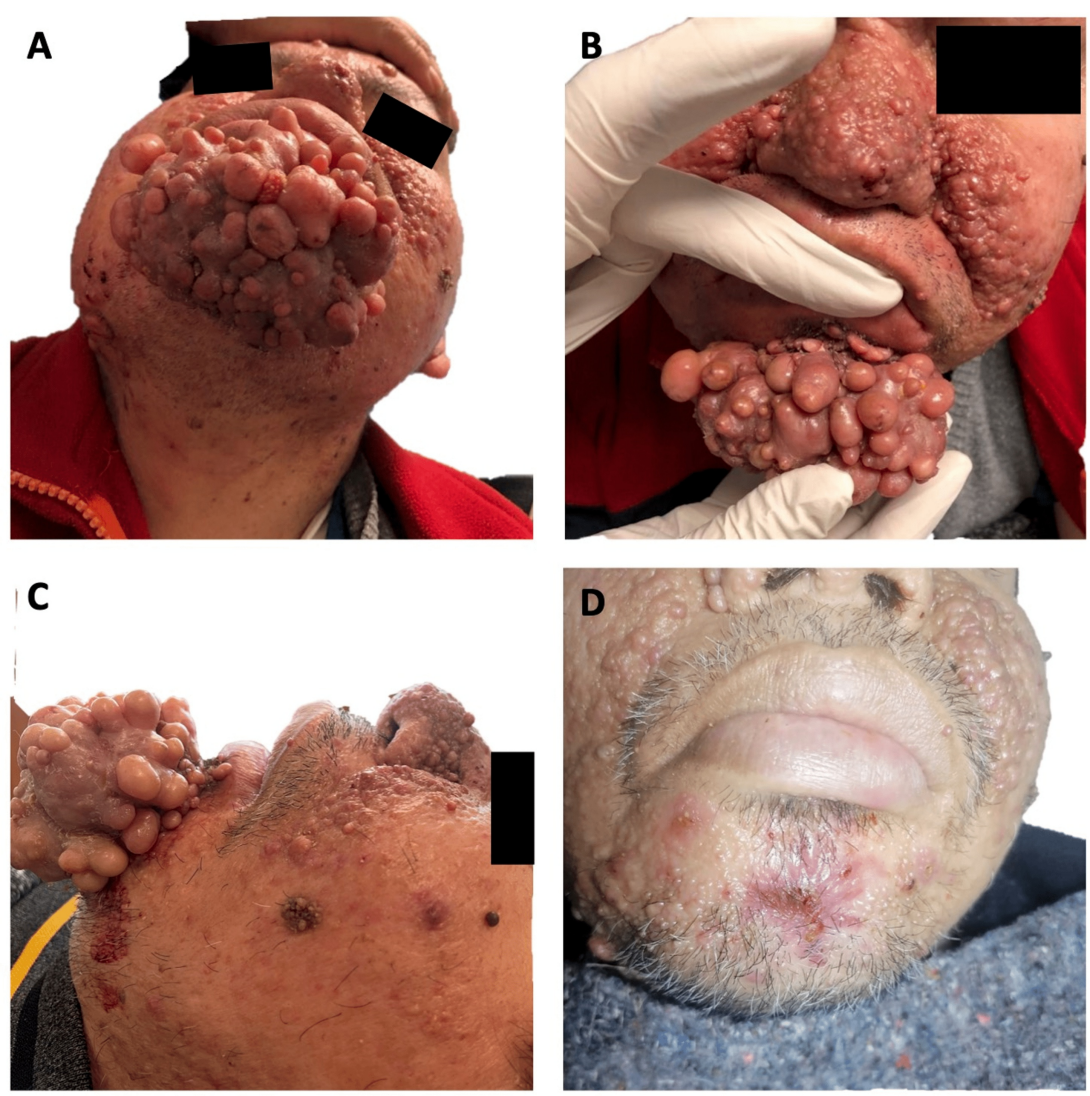
Clinical images of the patient’s gigantic angiofibroma A. Submandibular view showing multiple angiofibromas. B. View of the base of a prominent angiofibroma. C. Lateral view highlighting the angiofibroma’s tendency to bleed. D. Post-resection photo of the patient.

He was brought to the consultation by his sister, who reported an erythematous pink, well-defined nodular neoformation on the chin that bled easily upon manipulation. The size of the growth made it difficult for the patient to chew, perform dental hygiene, and undergo dental extractions. Three years ago, the neoformation started out as a singular small growth that grew slowly. Three months prior to consultation, the neoformation grew rapidly to 10x7x5 cm, comprised of approximately 35 confluent dome-shaped nodular growths fused into one mass (Figure 1A, 1B, 1C). The consistency of the growth was firm, but not hard or calcified. Surgical resection was performed under xylocaine with epinephrine and electric cauterization for hemostasis (Figure 1D).

**Figure 2:**
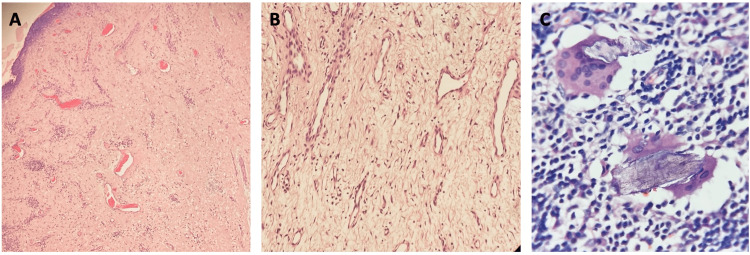
Histological images of the gigantic angiofibroma A. H&E, 10×. A non-encapsulated neoformation is seen extending from the papillary dermis to the reticular dermis, composed of giant fusiform cells with angulated, star-shaped nuclei. Several vascular lumens lined by a single layer of epithelial cells are present within a collagenous stroma. B. H&E, 20×. Loose stroma surrounding vascular lumens lined by a single layer of epithelial cells. C. H&E, 40×. Inflammatory infiltrate consisting of lymphocytes, plasma cells, and foreign body-type multinucleated giant cells surrounding free hairs.

The neoformation was cut longitudinally and stained with H&E. On examination, a non-encapsulated neoformation was observed between the papillary and reticular dermis, composed of large fusiform cells with angulated, star-shaped nuclei, intermixed with lymphocytes, plasma cells, and multinucleated giant cells (Figures 2A, 2B). The giant multinucleated cells presented a foreign body reaction, surrounding free hairs. The stroma was loose in some areas and collagenous in others and displayed multiple vascular lumens wrapped in a single layer of endothelial cells. The overall histological diagnosis was an inflamed angiofibroma with a granulomatous foreign body reaction, as seen in Figure 2C.

## Discussion

Facial angiofibroma is one of the most common cutaneous lesions in TSC and is included in the diagnostic criteria; however, giant angiofibromas are extremely rare. Under the microscope, they look like collections of stellate cells and blood vessels. Their mechanism of development is not well known. TSC is related to the dysregulation of the mTOR pathway, which is hypothesized to contribute to the overgrowth of angiofibromas [3]. There are few case reports of giant angiofibroma. In 1964, Johnson reported the first case of a cauliflower-like giant angiofibroma, which was surgically removed in a patient with chronic grand mal seizures [4]. Wataya-Kaneda et al. (1996) reported a large facial giant angiofibroma; karyotype analysis revealed a chromosomal translocation involving 12q and 15q [5]. The tumors on the patient’s nose and cheeks were resected below the subdermal fat level.

Kacerovska et al. reported two cases in Switzerland in which angiofibromas grew over a period of 15 years in one case and over 60 years in the other [6]. Both time frames reflect much slower progression compared to our case, which involved three years of slow growth followed by three months of rapid enlargement. Histologically, they identified numerous large, dilated vessels that stained positive for lymphatic markers. These vessels were observed microscopically and interpreted as evidence of lymphostasis, likely secondary to lymphedema. The authors proposed that lymphedema may play a role in the formation of giant angiofibromas [6].

An unusual histological finding in our case is the giant multinucleated cells, which are not common among TSC-associated or solitary angiofibromas. Kacerovska et al. also found multinucleated floret-like giant cells in their sample, which they attributed to lymphedema [6]. We propose that another growth mechanism for giant angiofibroma is rapid enlargement caused by a foreign body reaction superimposed on the underlying TSC mTOR pathway-related dysregulation. The foreign body reaction is likely due to naked hairs and follicular disruption. Figure 2 shows three histological images of the angiofibroma, with Figure 2C illustrating the foreign body reaction.

Table 1 summarizes case reports of angiofibromas measuring ≥2 cm from the literature review. The differential diagnosis for angiofibromas includes trichodiscomas and fibrofolliculomas. Given their largely benign clinical course, management decisions should be focused on managing any more symptoms caused by the size of the angiofibroma. These may include difficulty performing oral hygiene or eating, obstruction of nasal passages, vision obstruction, or psychosocial distress. The most effective treatment of any facial angiofibroma will be surgical resection. Topical sirolimus can be considered post-surgery to help prevent recurrence. For smaller angiofibromas, topical sirolimus (mTOR inhibitor) may provide regression and prevent growth in patients where the risk-benefit of surgical resection does not favor surgery. A study of 33 TSC patients with facial angiofibromas who received topical sirolimus found a statistically significant increase in health-related quality of life [7].

**Table 1:** Reported cases of angiofibromas measuring ≥2 cm #q indicates the chromosome number and the long arm of the chromosome. NR, not reported

Investigator (year)	Growth time	Size	Description
Butterworth and Wilson (1941) [8]	NR	4.5x9 cm	20-year-old man with a brownish-red, well-defined plaque on the right temporal region, with "excessive" lanugo hair
	20 years	2x2x4 cm	22-year-old man with a yellowish-red, inverted triangular plaque external to the left eye
	20 years	2x3.5 cm	22-year-old woman with a smooth, firm, well-demarcated raised pink plaque
	10 years	2x4.5 cm	11-year-old boy with a slightly infiltrated yellowish-brown plaque with irregular borders in front of the left ear
	13 years	1.2x2 cm	13-year-old girl with four superficial, infiltrated, dull red plaques on the right cheek and left forehead
Johnson (1964) [4]	21 years	7.5x17.5x3.75 cm	24-year-old woman with a cauliflower- or raspberry-like growth over the malar region, and a fibromatous, shiny, elevated plaque-like lesion on the forehead and left eyelid
Willis and Garcia (1978) [9]	10 years	6.5x5 cm	20-year-old man with a brownish-red plaque on the left malar area with numerous papules
Wataya-Kaneda et. al. (1997) [5]	27 years	0.5-2 cm 4x1 cm 2x1 cm	28-year-old Japanese woman with numerous confluent reddish nodules forming large plaques covering the central part of her face 2 additional dark-red plaques on right jaw and forehead. 12q to 15q translation in tumor cells
Kacerovska et al. (2012) [6]	15 years	0.5-2 cm (~6-8 cm together)	29-year-old European woman with multiple confluent large nodules on the chin, nasolabial folds, and cheeks
	60 years	NR	66-year-old European woman with four confluent medium-sized nasal nodules obstructing the left nostril
Moore et. al. (2023)	3 years	10x7x5 cm	47-year-old Hispanic man with 35 large confluent nodules on the mandible

## Conclusions

Due to the extremely infrequent nature of giant angiofibromas, large studies to compare findings are difficult to execute. Our case demonstrates that angiofibromas are not merely a cosmetic concern but can significantly impact quality of life and function. Surgical resection under local anesthesia is feasible regardless of lesion size. The growth pattern observed in this case warrants annual to biannual follow-up of such lesions. A possible mechanism for giant angiofibroma formation may involve rapid enlargement due to a foreign body reaction superimposed on the underlying mTORopathy. Further case reports with detailed histological findings are needed to advance these evolving theories.

## References

[1] Islam MP (2021). Tuberous sclerosis complex. Semin Pediatr Neurol.

[2] Randle SC (2017). Tuberous sclerosis complex: a review. Pediatr Ann.

[3] Boronat S, Barber I (2018). Less common manifestations in TSC. Am J Med Genet C Semin Med Genet.

[4] Johnson SA (1964). Adenoma sebaceum showing a cauliflower-like growth. Arch Dermatol.

[5] Wataya-Kaneda M, Yano K, Hosokawa K, Yoshikawa K (1997). A case of tuberous sclerosis with a giant angiofibroma. J Dermatol.

[6] Kacerovska D, Kerl K, Michal M (2012). Giant angiofibromas in tuberous sclerosis complex: a possible role for localized lymphedema in their pathogenesis. J Am Acad Dermatol.

[7] Hatano T, Ohno Y, Imai Y, Moritake J, Endo K, Tamari M, Egawa S (2020). Improved health-related quality of life in patients treated with topical sirolimus for facial angiofibroma associated with tuberous sclerosis complex. Orphanet J Rare Dis.

[8] Butterworth T (1941). Dermatologic aspects of tuberous sclerosis. Arch Dermatol.

[9] Willis WF, Garcia RL (1978). Giant angiofibroma in tuberous sclerosis. Arch Dermatol.

